# Supplementary material for: On the Ionic Conductivity of Cation Exchange Membranes in Mixed Sulfates Using the Two-Phase Model
Source: Membranes (Basel). 2023 Sep 26;13(10):811. doi: 10.3390/membranes13100811 (PMC10608779; doi:10.3390/membranes13100811)
Supplement: Supplementary file 1 [file membranes-13-00811-s001.zip › membranes-2578191-supplementary.pdf]

membranes

Supporting information

On the ionic conductivity of cation exchange membranes in mixed sulfates using the two-phase model

Liansheng Wu †, Haodong Jiang †, Tao Luo * and Xinlong Wang

Ministry of Education`s Research Center for Comprehensive Utilization and Clean Process Engineering of Phosphorous Resources, School of Chemical Engineering, Sichuan University, Chengdu 610065, China; liansheng_wu@163.com (L.W.); 15310838208@163.com (H.J.); wangxl@scu.edu.cn (X.W.)

* Correspondence: tao.luo@scu.edu.cn

† These authors contributed equally to this work.

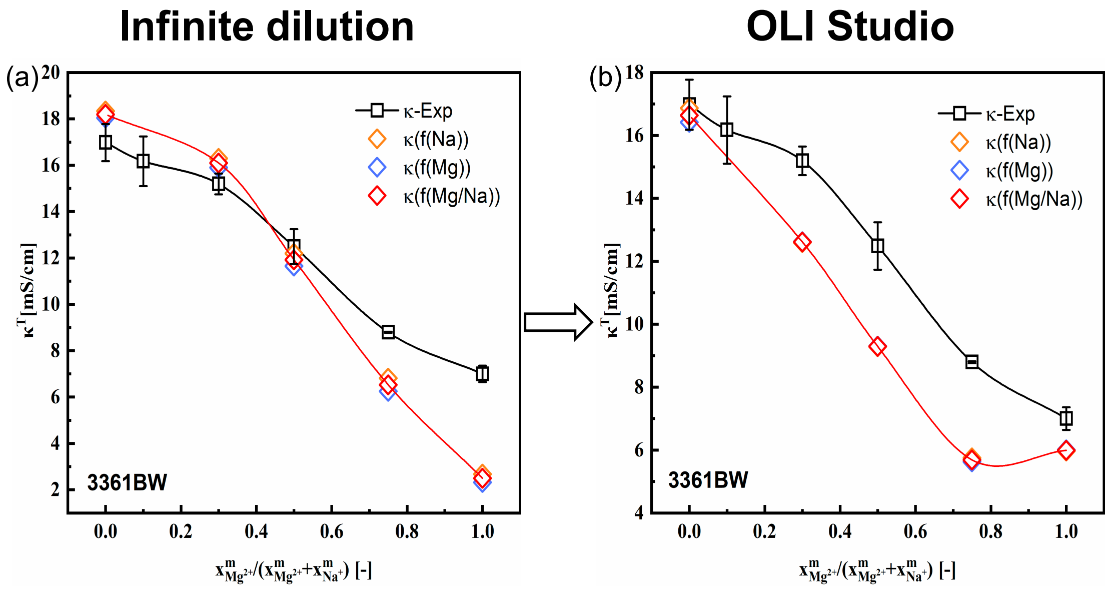

Figure S1. Comparation of the total conductivity variations for the 3361BW membrane in mixed $MgSO_4$+Na_2SO_4 electrolytes as a function of the cation composition in the external solution. The inter-gel phase conductivity (κ^{in}) is obtained via two different approaches: calculated with (**a**) the ionic mobilities in infinite dilution, (**b**) with the inter-gel phase conductivity data simulated in the OLI Studio.

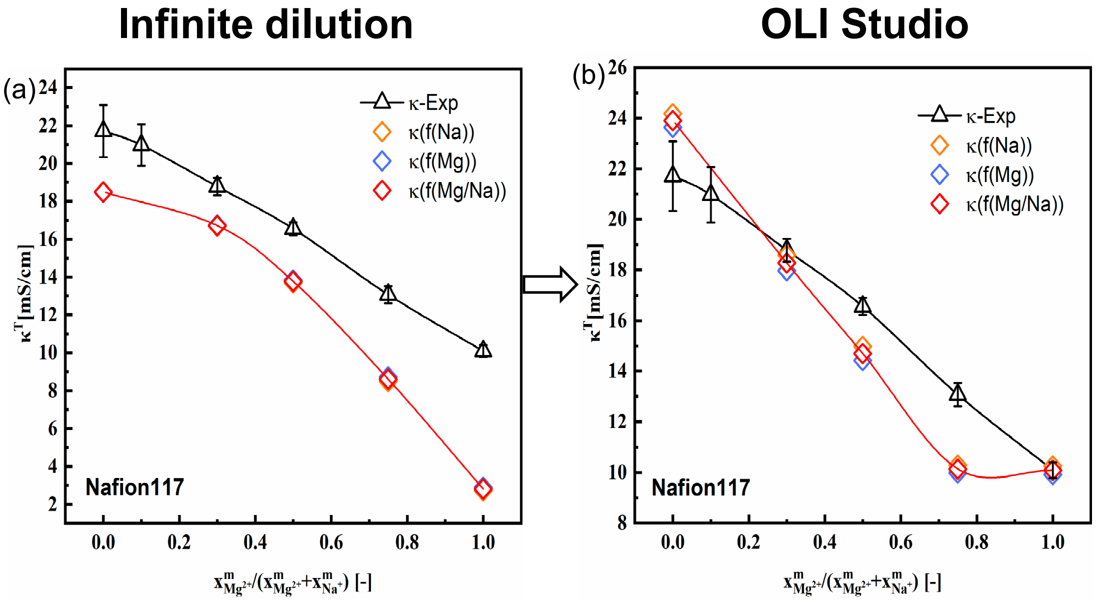

Figure S2. Comparation of the total conductivity variations for the Nafion117 membrane in mixed $MgSO_4$+Na_2SO_4 electrolytes as a function of the cation composition in the external solution. The inter-gel phase conductivity (κ^{in}) is obtained via two different approaches: calculated with (**a**) the ionic mobilities in infinite dilution, (**b**) with the inter-gel phase conductivity data simulated in the OLI Studio.

Table S1 ~ **Table S8** list the numerical results of the calculated total conductivity for three CEMs in equilibrium with mixed sulfates of different total sulfate concentration (0.1, 0.5, and 1.0 M) in this work.

Table S1. The numerical results of CEMs' total conductivity under mixed $MgSO_4$+Na_2SO_4 electrolytes of different total sulfate concentration. The inter-gel phase conductivity (κ^{in}) values are obtained from the ionic mobilities in infinite dilution.

Membrane	SO_4^{2-} Con. [M]	Equivalent Mg^{2+} ratio in the external solution [-]	*κ*-total [mS cm⁻¹]			
			Exp.	f (Na)	f (Mg)	f (Mg/Na)
**3361BW**	**0.1**	0	12.36	12.55	13.58	13.06
		0.1	10.23	-	-	-
		0.3	8.05	9.48	10.01	9.74
		0.5	6.46	6.88	7.05	6.96
		0.75	5.24	4.60	4.58	4.59
		1.0	4.37	1.23	1.30	1.27
	0.5	0	16.98	18.34	18.04	18.19
		0.1	16.17	-	-	-
		0.3	15.19	16.30	15.90	16.09
		0.5	12.49	12.20	11.66	11.92
		0.75	8.79	6.81	6.25	6.53
		1.0	7.00	2.68	2.32	2.49
	1.0	0	20.74	21.25	20.14	20.69
		0.1	18.98	-	-	-
		0.3	18.71	20.20	19.03	19.61
		0.5	15.54	15.55	14.39	14.96
		0.75	11.22	8.42	7.52	7.96
		1.0	8.28	3.12	2.60	2.85
**Membrane**			***κ*-total [mS cm⁻¹]**			

	SO_4^{2-} Con. [M]	Equivalent Mg^{2+} ratio in the external solution [-]	Exp.	f (Na)	f (Mg)	f (Mg/Na)
CSE	0.1	0	12.36	8.32	8.89	8.60
		0.1	10.23	9.26	10.17	9.71
		0.3	8.053	11.67	12.65	12.15
		0.5	6.46	12.30	13.56	12.91
		0.75	5.24	13.07	14.50	13.77
		1.0	4.37	10.99	13.36	12.12
	0.5	0	16.98	9.96	9.97	9.97
		0.1	16.17	10.18	10.12	10.15
		0.3	15.19	11.83	11.82	11.82
		0.5	12.49	13.73	13.85	13.79
		0.75	8.79	15.56	15.89	15.72
		1.0	7.00	16.60	17.39	16.99
	1.0	0	20.74	11.15	10.72	10.93
		0.1	18.98	11.63	11.09	11.35
		0.3	18.71	12.95	12.46	12.70
		0.5	15.54	14.66	14.24	14.45
		0.75	11.22	16.92	16.66	16.79
		1.0	8.28	19.20	19.09	19.15

Membrane	SO_4^{2-} Con. [M]	Equivalent Mg^{2+} ratio in the external solution [-]	κ-total [mS cm^{-1}]			
			Exp.	f (Na)	f (Mg)	f (Mg/Na)
Nafion117	0.1	0	12.36	14.64	13.69	14.16
		0.1	10.23	13.31	12.59	12.94
		0.3	8.053	10.38	9.96	10.17
		0.5	6.46	7.97	7.77	7.87
		0.75	5.24	5.23	5.20	5.21
		1.0	4.36	2.29	2.329	2.309
	0.5	0	16.98	18.51	18.48	18.498
		0.1	16.178	-	-	-
		0.3	15.19	16.69	16.74	16.72
		0.5	12.48	13.71	13.84	13.78
		0.75	8.79	8.48	8.72	8.60
		1.0	7.00	2.72	2.90	2.81
	1.0	0	20.74	20.08	20.51	20.29
		0.1	18.98	19.56	19.99	19.77
		0.3	18.71	17.57	17.97	17.77
		0.5	15.54	14.81	15.23	15.02
		0.75	11.22	9.37	9.77	9.57
		1.0	8.28	2.89	3.13	3.01

Table S2. The numerical results of CEMs` total conductivity under mixed H_2SO_4 + Na_2SO_4 electrolytes of different total sulfate concentration. The inter-gel phase conductivity (κ^{in}) values are obtained from the ionic mobilities in infinite dilution.

Membrane	SO_4^{2-} Con. [M]	Equivalent H^+ ratio in the external solution [-]	κ-total [mS cm^{-1}]			
			Exp.	f(Na)	f(H)	f(H/Na)
**3361BW**	**0.1**	0	12.56	13.11	10.20	11.56
		0.1	13.89	-	-	-
		0.3	16.54	16.18	13.77	14.93
		0.5	22.15	29.59	22.69	25.91
		0.75	30.22	46.78	30.02	37.48

		1.0	45.75	94.01	60.61	75.48
	0.5	0	14.21	17.11	16.88	16.99
		0.1	18.68	-	-	-
		0.3	23.04	22.06	22.10	22.08
		0.5	27.33	37.80	33.42	35.54
		0.75	53.77	61.78	55.44	58.53
		1.0	98.20	126.94	106.97	116.52
	1.0	0	15.15	21.21	25.36	23.19
		0.1	21.18	-	-	-
		0.3	26.10	-	-	-
		0.5	33.60	39.75	42.64	41.17
		0.75	60.40	77.44	76.18	76.80
		1.0	116.86	147.32	141.78	144.52

Membrane	SO_4^{2-} Con. [M]	Equivalent H^+ ratio in the external solution [-]	*κ*-total [mS cm^{-1}]			
			Exp.	f(Na)	f(H)	f(H/Na)
CSE	0.1	0	12.56	7.60	7.64	7.62
		0.1	13.89	11.33	11.37	11.35
		0.3	16.54	13.81	13.88	13.85
		0.5	22.15	19.37	19.48	19.42
		0.75	30.22	33.95	34.08	34.02
		1.0	45.75	45.52	45.87	45.69
	0.5	0	14.21	10.42	10.41	10.41
		0.1	18.68	16.62	16.61	16.61
		0.3	23.04	19.34	19.33	19.34
		0.5	27.33	23.46	23.50	23.48
		0.75	53.77	33.47	33.49	33.48
		1.0	98.20	57.77	57.94	57.86
	1.0	0	15.15	10.82	10.81	10.81
		0.1	21.18	13.64	13.62	13.63
		0.3	26.10	17.82	17.80	17.81
		0.5	33.60	25.36	25.34	25.35
		0.75	60.40	40.49	40.50	40.49
		1.0	116.86	72.06	71.97	72.02

Membrane	SO_4^{2-} Con. [M]	Equivalent H^+ ratio in the external solution [-]	*κ*-total [mS cm^{-1}]			
			Exp.	f(Na)	f(H)	f(H/Na)
Nafion117	0.1	0	12.56	14.17	15.26	14.71
		0.1	13.89	27.21	29.29	28.23
		0.3	16.54	31.77	34.29	33.01
		0.5	22.15	34.28	37.29	35.75
		0.75	30.22	45.94	49.77	47.82
		1.0	45.75	61.95	67.09	64.47
	0.5	0	14.21	17.96	17.96	17.96
		0.1	18.68	30.44	31.81	31.12
		0.3	23.04	36.73	37.32	37.02
		0.5	27.33	43.99	44.62	44.30
		0.75	53.77	56.28	57.41	56.84
		1.0	98.20	72.34	74.66	73.49
	1.0	0	15.15	19.51	19.01	19.26
		0.1	21.18	31.01	30.48	30.74
		0.3	26.10	39.39	38.48	38.94
		0.5	33.60	38.83	37.75	38.29
		0.75	60.40	58.87	57.03	57.94
		1.0	116.86	86.33	84.33	85.32

Table S3. The numerical results of CEMs' total conductivity under mixed $MgSO_4$ + Na_2SO_4 electrolytes of different total sulfate concentration. The inter-gel phase conductivity (κ^{in}) values are simply equivalent to the conductivity of the external solution (κ^{s}).

**Membrane**	**SO_4^{2-} Con. [M]**	**Equivalent Mg^{2+} ratio in the external solution [-]**	***κ*-total [mS cm^{-1}]**			
			**Exp.**	**f(Na)**	**f(Mg)**	**f(Mg/Na)**
**3361BW**	**0.1**	0	12.36	16.91	16.79	16.85
		0.1	10.23	13.74	14.04	13.88
		0.3	8.05	10.48	11.01	10.74
		0.5	6.46	7.90	8.57	8.23
		0.75	5.24	7.02	7.62	7.31
		1.0	4.37	8.57	8.82	8.70
	0.5	0	16.98	23.44	28.06	25.65
		0.1	16.17	-	-	-
		0.3	15.19	19.10	22.89	20.91
		0.5	12.49	14.77	18.06	16.33
		0.75	8.79	9.70	12.56	11.03
		1.0	7.00	10.31	13.01	11.58
	1.0	0	20.74	29.92	37.37	33.44
		0.1	18.98	-	-	-
		0.3	18.71	24.72	31.08	27.72
		0.5	15.54	19.39	24.86	21.96
		0.75	11.22	13.07	17.60	15.17
		1.0	8.28	12.70	16.73	14.58

**Membrane**	**SO_4^{2-} Con. [M]**	**Equivalent Mg^{2+} ratio in the external solution [-]**	***κ*-total [mS cm^{-1}]**			
			**Exp.**	**f(Na)**	**f(Mg)**	**f(Mg/Na)**
CSE	0.1	0	12.36	11.57	11.81	11.69
		0.1	10.23	8.28	8.59	8.44
		0.3	8.05	4.89	5.19	5.04
		0.5	6.46	3.74	4.01	3.87
		0.75	5.24	3.42	3.66	3.54
		1.0	4.37	3.96	4.18	4.07
	0.5	0	16.98	15.39	16.66	16.02
		0.1	16.17	13.45	14.62	14.03
		0.3	15.19	9.58	10.54	10.05
		0.5	12.49	6.77	7.53	7.14
		0.75	8.79	4.36	4.94	4.64
		1.0	7.00	4.58	5.16	4.86
	1.0	0	20.74	17.32	19.13	18.20
		0.1	18.98	14.11	15.73	14.90
		0.3	18.71	11.67	13.07	12.35
		0.5	15.54	8.29	9.40	8.83
		0.75	11.22	5.01	5.80	5.39
		1.0	8.28	4.29	4.97	4.61

**Membrane**	**SO_4^{2-} Con. [M]**	**Equivalent Mg^{2+} ratio in the external solution [-]**	***κ*-total [mS cm^{-1}]**			
			**Exp.**	**f(Na)**	**f(Mg)**	**f(Mg/Na)**
Nafion117	0.1	0	12.36	24.83	24.73	24.78
		0.1	10.23	20.43	20.38	20.41
		0.3	8.05	15.29	15.27	15.28
		0.5	6.46	12.25	12.25	12.25

		0.75	5.24	10.88	10.87	10.88
		1.0	4.37	10.34	10.33	10.34
	0.5	0	16.98	29.71	29.90	29.81
		0.1	16.17	-	-	-
		0.3	15.19	23.65	23.81	23.73
		0.5	12.49	19.44	19.58	19.51
		0.75	8.79	13.72	13.85	13.78
		1.0	7.00	13.63	13.74	13.69
	1.0	0	20.74	27.00	27.31	27.16
		0.1	18.98	26.11	26.42	26.26
		0.3	18.71	24.08	24.36	24.22
		0.5	15.54	20.64	20.87	20.76
		0.75	11.22	15.77	15.96	15.86
		1.0	8.28	14.44	14.62	14.53

Table S4. The numerical results of CEMs` total conductivity under mixed H_2SO_4 + Na_2SO_4 electrolytes of different total sulfate concentration. The inter-gel phase conductivity (κ^{in}) values are simply equivalent to the conductivity of the external solution (κ^{s}).

Membrane	SO_4^{2-} Con. [M]	Equivalent H^+ ratio in the external solution [-]	κ-total [mS cm^{-1}]			
			Exp.	f(Na)	f(H)	f(H/Na)
**3361BW**	**0.1**	0	12.56	17.27	17.04	17.16
		0.1	13.89	-	-	-
		0.3	16.54	18.74	19.65	19.19
		0.5	22.15	26.69	27.17	26.93
		0.75	30.22	39.42	39.19	39.30
		1.0	45.75	61.51	58.57	60.02
	0.5	0	14.21	24.54	30.23	27.24
		0.1	18.68	-	-	-
		0.3	23.04	34.50	42.63	38.35
		0.5	27.33	43.44	53.92	48.40
		0.75	53.77	80.40	93.97	86.92
		1.0	98.20	150.64	163.92	157.14
	1.0	0	15.15	26.33	35.63	30.63
		0.1	21.18	-	-	-
		0.3	26.10	43.39	57.40	49.91
		0.5	33.60	54.62	72.72	63.03
		0.75	60.40	94.81	122.07	107.58
		1.0	116.86	190.68	226.61	207.87
**Membrane**	**SO_4^{2-} Con. [M]**	**Equivalent H^+ ratio in the external solution [-]**	**κ-total [mS cm^{-1}]**			
			**Exp.**	**f(Na)**	**f(H)**	**f(H/Na)**
CSE	0.1	0	12.56	12.07	12.56	12.32
		0.1	13.89	7.77	8.69	8.21
		0.3	16.54	14.75	15.61	15.17
		0.5	22.15	22.51	23.25	22.88
		0.75	30.22	29.45	30.51	29.98
		1.0	45.75	60.69	59.20	59.94
	0.5	0	14.21	15.50	18.44	16.91
		0.1	18.68	12.38	15.43	13.82
		0.3	23.04	16.39	20.27	18.23
		0.5	27.33	28.22	33.59	30.79

		0.75	53.77	51.47	59.40	55.29
		1.0	98.20	104.43	114.72	109.45
	1.0	0	15.15	15.69	19.80	17.63
		0.1	21.18	16.31	20.90	18.46
		0.3	26.10	19.18	24.85	21.83
		0.5	33.60	27.76	35.45	31.37
		0.75	60.40	57.75	70.71	63.90
		1.0	116.86	90.91	110.06	100.03

**Membrane**	**SO_4^{2-}Con. [M]**	**Equivalent H^+ ratio in the external solution [-]**	**κ-total [mS cm⁻¹]**			
			**Exp.**	**f(Na)**	**f(H)**	**f(H/Na)**
Nafion117	0.1	0	12.56	25.17	25.87	25.52
		0.1	13.89	17.85	17.83	17.84
		0.3	16.54	27.53	27.87	27.70
		0.5	22.15	42.89	44.00	43.44
		0.75	30.22	65.87	68.16	67.00
		1.0	45.75	110.66	116.38	113.48
	0.5	0	14.21	25.91	24.61	25.25
		0.1	18.68	23.52	22.02	22.76
		0.3	23.04	27.51	25.65	26.57
		0.5	27.33	40.73	38.32	39.51
		0.75	53.77	71.88	68.50	70.17
		1.0	98.20	145.87	142.37	144.11
	1.0	0	15.15	28.08	26.04	27.04
		0.1	21.18	21.80	19.69	20.72
		0.3	26.10	28.43	25.70	27.03
		0.5	33.60	38.26	34.69	36.43
		0.75	60.40	69.02	63.28	66.09
		1.0	116.86	116.81	108.18	112.42

Table S5. The numerical results of CEMs' total conductivity under mixed $MgSO_4$ + Na_2SO_4 electrolytes of different total sulfate concentration. The inter-gel phase conductivity (κ^{in}) values are obtained from the conductivity data analyzed in the OLI Studio.

**Membrane**	**SO_4^{2-}Con. [M]**	**Equivalent Mg^{2+}ratio in the external solution [-]**	**κ-total [mS cm⁻¹]**			
			**Exp.**	**f(Na)**	**f(Mg)**	**f(Mg/Na)**
**3361BW**	**0.1**	0	12.36	10.79	12.62	11.67
		0.1	10.23	8.85	10.09	9.45
		0.3	8.05	6.48	7.34	6.89
		0.5	6.46	4.87	5.39	5.12
		0.75	5.24	4.27	4.74	4.50
		1.0	4.37	3.02	4.20	3.57
	0.5	0	16.98	16.87	16.42	16.64
		0.1	16.17	-	-	-
		0.3	15.19	12.60	12.62	12.61
		0.5	12.49	9.29	9.30	9.29
		0.75	8.79	5.75	5.63	5.69
		1.0	7.00	5.97	6.01	5.99
	1.0	0	20.74	21.28	20.12	20.70
		0.1	18.98	0.00	0.00	0.00
		0.3	18.71	16.51	15.87	16.19

		0.5	15.54	12.35	11.88	12.12
		0.75	11.22	7.70	7.32	7.51
		1.0	8.28	7.31	7.13	7.22

Membrane	SO_4^{2-} Con. [M]	Equivalent Mg^{2+} ratio in the external solution [-]	κ-total [mS cm^{-1}]			
			Exp.	f(Na)	f(Mg)	f(Mg/Na)
CSE	0.1	0	8.07	8.60	9.55	9.07
		0.1	5.64	5.68	6.38	6.02
		0.3	3.82	3.59	3.77	3.68
		0.5	3.04	2.55	2.73	2.64
		0.75	2.51	2.21	2.44	2.32
		1.0	2.07	1.80	2.38	2.07
	0.5	0	10.00	11.07	11.41	11.24
		0.1	9.22	9.74	9.93	9.83
		0.3	7.07	6.81	6.88	6.84
		0.5	5.42	4.69	4.71	4.70
		0.75	3.97	2.92	2.90	2.91
		1.0	2.88	2.81	2.94	2.88
	1.0	0	11.34	12.84	12.64	12.74
		0.1	9.75	10.67	10.26	10.47
		0.3	8.52	8.44	8.22	8.33
		0.5	6.54	5.84	5.66	5.75
		0.75	4.50	3.42	3.25	3.33
		1.0	2.81	2.87	2.75	2.81

Membrane	SO_4^{2-} Con. [M]	Equivalent Mg^{2+} ratio in the external solution [-]	κ-total [mS cm^{-1}]			
			Exp.	f(Na)	f(Mg)	f(Mg/Na)
Nafion117	0.1	0	17.19	20.08	17.81	18.91
		0.1	14.98	16.66	14.99	15.81
		0.3	12.11	12.31	11.18	11.74
		0.5	10.66	9.88	9.06	9.46
		0.75	9.00	8.82	8.11	8.46
		1.0	7.86	8.23	7.49	7.85
	0.5	0	21.70	24.18	23.64	23.91
		0.1	20.97	-	-	-
		0.3	18.78	18.59	17.97	18.28
		0.5	16.56	14.98	14.42	14.70
		0.75	13.08	10.29	9.99	10.13
		1.0	10.09	10.26	9.92	10.09
	1.0	0	19.98	21.37	21.69	21.53
		0.1	19.99	20.41	20.59	20.50
		0.3	19.33	18.28	18.18	18.23
		0.5	17.67	15.34	15.15	15.25
		0.75	14.82	11.40	11.26	11.33
		1.0	10.62	10.48	10.31	10.40

Table S6. The numerical results of CEMs′ total conductivity under mixed H_2SO_4 + Na_2SO_4 electrolytes of different total sulfate concentration. The inter-gel phase conductivity (κ^{in}) values are obtained from the conductivity data analyzed in the OLI Studio.

Membrane	SO_4^{2-} Con. [M]	Equivalent H^+ ratio in the external solution [-]	κ-total [mS cm^{-1}]			
			Exp.	f(Na)	f(H)	f(H/Na)
**3361BW**	**0.1**	0	12.56	13.07	10.77	11.87

		0.1	13.89	0.00	0.00	0.00
		0.3	16.54	14.16	12.52	13.32
		0.5	22.15	19.95	16.93	18.38
		0.75	30.22	25.83	19.59	22.50
		1.0	45.75	46.75	37.02	41.60
	0.5	0	14.21	16.02	15.63	15.82
		0.1	18.68	0.00	0.00	0.00
		0.3	23.04	21.14	19.89	20.50
		0.5	27.33	24.93	22.63	23.75
		0.75	53.77	51.40	46.50	48.89
		1.0	98.20	105.46	92.90	98.98
	1.0	0	15.15	18.34	20.87	19.56
		0.1	21.18	0.00	0.00	0.00
		0.3	26.10	25.96	26.09	26.03
		0.5	33.60	31.64	31.38	31.51
		0.75	60.40	56.96	55.59	56.27
		1.0	116.86	131.11	126.78	128.92
**Membrane**	**SO_4^{2-} Con. [M]**	**Equivalent H^+ ratio in the external solution [-]**	***κ*-total [mS cm^{-1}]**			
			**Exp.**	**f(Na)**	**f(H)**	**f(H/Na)**
CSE	0.1	0	7.25	7.87	8.69	8.27
		0.1	8.22	6.13	6.24	6.18
		0.3	11.53	9.91	10.78	10.34
		0.5	16.00	14.80	16.35	15.56
		0.75	23.30	23.54	24.65	24.09
		1.0	35.41	37.67	43.25	40.36
	0.5	0	10.42	11.87	11.88	11.88
		0.1	11.31	10.25	9.86	10.06
		0.3	14.12	12.85	12.58	12.71
		0.5	19.52	18.20	19.07	18.63
		0.75	37.03	36.82	38.00	37.40
		1.0	61.84	67.79	73.27	70.48
	1.0	0	9.82	11.22	11.17	11.19
		0.1	11.65	11.49	11.41	11.45
		0.3	14.56	13.46	13.32	13.39
		0.5	20.01	18.87	18.95	18.91
		0.75	39.65	39.32	40.15	39.73
		1.0	63.29	72.78	71.48	72.13
**Membrane**	**SO_4^{2-} Con. [M]**	**Equivalent H^+ ratio in the external solution [-]**	***κ*-total [mS cm^{-1}]**			
			**Exp.**	**f(Na)**	**f(H)**	**f(H/Na)**
Nafion117	0.1	0	14.83	20.38	22.93	21.62
		0.1	17.10	14.63	15.76	15.19
		0.3	21.28	22.32	24.59	23.43
		0.5	28.51	33.83	38.34	36.01
		0.75	42.00	53.63	60.76	57.08
		1.0	66.28	93.27	106.55	99.69
	0.5	0	17.96	20.91	21.19	21.05
		0.1	20.68	16.73	17.46	17.09
		0.3	22.71	20.99	21.20	21.10
		0.5	31.93	31.38	31.97	31.68
		0.75	51.04	55.28	57.33	56.29
		1.0	87.76	111.74	119.83	115.71
	1.0	0	20.07	22.38	22.07	22.22
		0.1	20.64	16.83	16.22	16.52

	0.3	26.42	22.26	21.37	21.81
	0.5	33.11	29.67	28.69	29.18
	0.75	57.80	56.11	54.10	55.10
	1.0	87.21	95.50	93.12	94.30

Table S7. The numerical fitting results of the correlation between κ^g and σ for CEMs in mixed $MgSO_4$ + Na_2SO_4 electrolytes, according to Equation 6.

**Membranes**	**Sulfate concentration [M]**	**σ [-]**	R^2 **[-]**
3361BW	0.1	14.497	0.900
	0.5	-3.780	0.995
	1.0	-2.017	0.999
CSE	0.1	5.219	0.972
	0.5	4.086	0.988
	1.0	3.514	0.972
Nafion117	0.1	6.276	0.999
	0.5	0.751	0.999
	1.0	-6.409	0.995

Table S8. The numerical fitting results of the correlation between κ^g and σ for CEMs in mixed H_2SO_4 + Na_2SO_4 electrolytes, according to Equation 6.

**Membranes**	**Sulfate concentration [M]**	**σ [-]**	R^2 **[-]**
3361BW	0.1	-33.601	0.975
	0.5	-21.728	0.987
	1.0	-23.994	0.999
CSE	0.1	6.937	0.976
	0.5	11.720	0.981
	1.0	-4.427	0.976
Nafion117	0.1	37.128	0.994
	0.5	55.350	0.994
	1.0	22.154	0.974

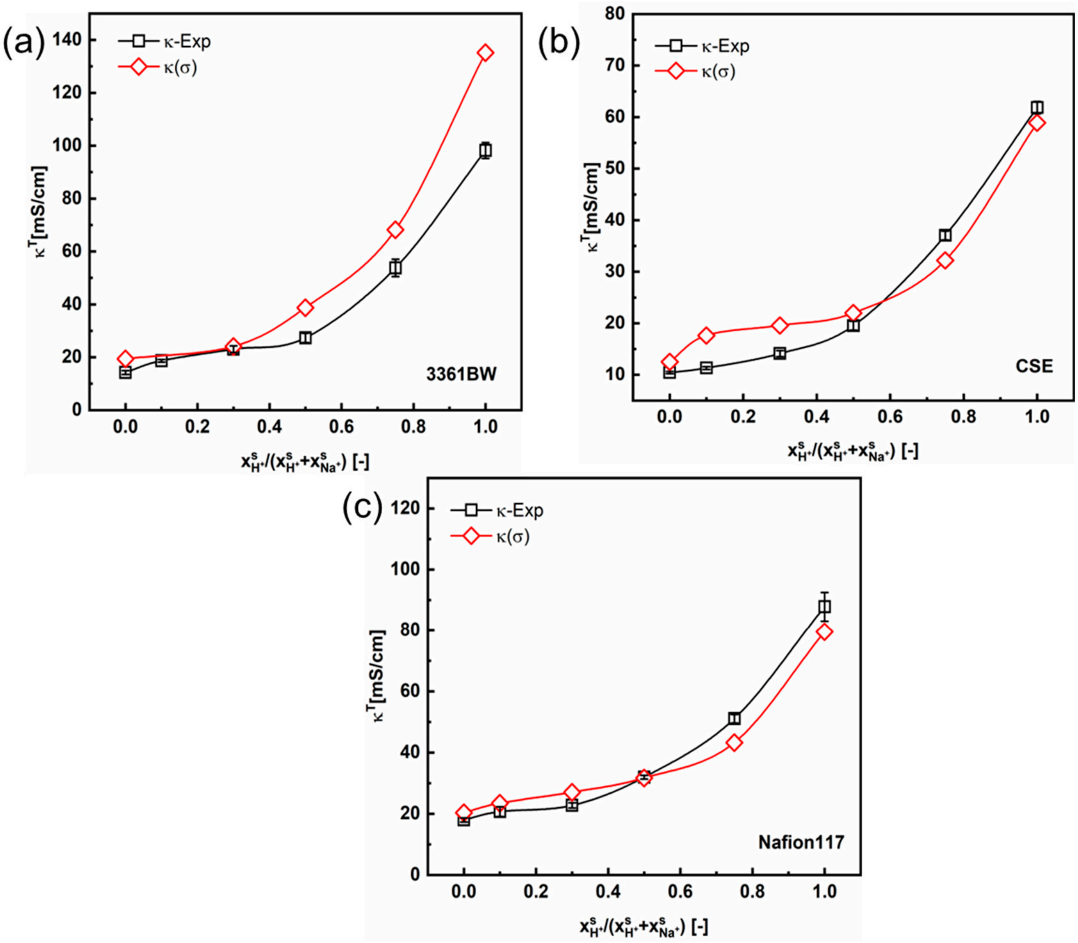

Figure S3. The variation of the experimental and the predicted total membrane conductivities with the cation composition of the mixed external $H_2SO_4 + Na_2SO_4$ electrolytes (total sulfate concentration 0.5 M). The gel phase conductivity (κ^g) of the CEMs is obtained from the nonlinear correlation method considering the interaction parameter (α).
